# Evaluation of cattle sub-species and growth-promoting technology on growth performance, carcass characteristics, and gas flux of steers finished in summer feedlot conditions

**DOI:** 10.1093/jas/skaf322

**Published:** 2025-09-24

**Authors:** Ashley K Schilling-Hazlett, Kimberly R Stackhouse-Lawson, Tony C Bryant, Juan J Vargas, Sara E Place, John P Ritten, Edilane C Martins, Willian A Souza, Maya A Swenson, Erin N Burke, Rhyse K Campion, Anna M Shadbolt, Pedro H V Carvalho

**Affiliations:** CSU AgNext, Department of Animal Sciences, Colorado State University, Fort Collins, CO 80523; CSU AgNext, Department of Animal Sciences, Colorado State University, Fort Collins, CO 80523; Five Rivers Cattle Feeding, Johnstown, CO 80534; CSU AgNext, Department of Animal Sciences, Colorado State University, Fort Collins, CO 80523; CSU AgNext, Department of Animal Sciences, Colorado State University, Fort Collins, CO 80523; CSU AgNext, Department of Animal Sciences, Colorado State University, Fort Collins, CO 80523; CSU AgNext, Department of Animal Sciences, Colorado State University, Fort Collins, CO 80523; CSU AgNext, Department of Animal Sciences, Colorado State University, Fort Collins, CO 80523; CSU AgNext, Department of Animal Sciences, Colorado State University, Fort Collins, CO 80523; CSU AgNext, Department of Animal Sciences, Colorado State University, Fort Collins, CO 80523; CSU AgNext, Department of Animal Sciences, Colorado State University, Fort Collins, CO 80523; CSU AgNext, Department of Animal Sciences, Colorado State University, Fort Collins, CO 80523; CSU AgNext, Department of Animal Sciences, Colorado State University, Fort Collins, CO 80523

**Keywords:** *Bos indicus*, *Bos taurus*, gas flux, growth performance, growth-promoting technology, sustainability

## Abstract

The objectives of this study were to evaluate the comparative growth performance, carcass characteristics, and gas flux of yearling *Bos indicus* (BI; Brahman) and *Bos taurus* (BT; Angus) steers managed with (TRT) and without (CON) the use of growth-promoting technology (GPT). One hundred BI (initial body weight (IBW) = 342 ± 31 kg) and 100 BT (IBW = 341 ± 21 kg) steers were fed for 180d in 2 consecutive phases. In Phase 1, d 0–83, cattle of each sub-species were blocked by body weight and randomly assigned to a management treatment in 10-hd research pens (5 pens/treatment). In Phase 2, d 84–180, cattle were moved and randomly assigned to a 50-hd research pen (1 pen/treatment) equipped with 1 GreenFeed automated head chamber system (C-Lock, Rapid City, SD, USA) and 5 SmartFeed bunk systems (C-Lock, Rapid City, SD, USA) for measuring individual gas flux of methane (CH_4_), carbon dioxide (CO_2_), oxygen (O_2_), and hydrogen (H_2_) and feed intake, respectively. Data were analyzed with R (R Core Team, 2021, v. 4.4.1) software to assess the fixed effects of cattle sub-species, treatment, and their interaction for growth performance collected in Phase 1 and 2, and gas flux which was only measured in Phase 2. In Phase 1, IBW did not differ (*P* > 0.75) by sub-species or treatment. Dry matter intake (DMI), average daily gain (ADG), and feed efficiency (G:F) were greater (*P* < 0.01) for BT, resulting in greater final body weight (FBW) for BT compared to BI. Within sub-species, DMI did not differ (*P* > 0.35) between treatments in Phase 1, but ADG, G:F, and FBW were greater (*P* < 0.01) for TRT compared to CON. In Phase 2, DMI increased with the use of GPT. However, there was a sub-species × treatment interaction (*P* ≤ 0.04) where greater increases in ADG and FBW were observed between TRT and CON for BT relative to BI. Furthermore, BI had a greater proportion of Standard and Select quality grades relative to BT. Daily CH_4_ production (g CH_4_/d), CH_4_ yield (g CH_4_/kg DMI), and yield of CH_4_ (% of gross energy intake) were less (*P* < 0.01) for BI than BT. The use of GPT decreased emissions intensity per unit ADG and carcass gain; furthermore, a sub-species × treatment interaction existed (*P* ≤ 0.05), where a greater decrease in EI was observed between CON and TRT for BT when compared to BI. Ultimately, BT had greater growth performance and carcass quality, but BI emitted less CH_4_, highlighting the complex tradeoffs resulting from sustainability-related research in beef production systems.

## Teaser

As greenhouse gases accumulate in the atmosphere at an accelerated rate, contributing to global warming, all supply chains will investigate innovations to improve climate adaptivity and resiliency. In the beef industry specifically, the use of growth-promoting technologies and cattle sub-species selection are examples of proposed innovations to improve the adaptive capacity and resiliency of the beef supply chain to ensure food security for current and future generations.

## Introduction

Sustainability is a dynamic state of continuous improvement that encapsulates balancing economic, social, and environmental factors in a constantly evolving ecosystem. As atmospheric concentrations of greenhouse gases (GHG) continue to increase at an unprecedented rate, resulting in increasingly severe, interconnected, and often irreversible climate impacts, a shift of perspective to mitigation in tandem with adaptation is required (Pörtner et al., 2022). When considering the agriculture industry, GHG are emitted from agricultural activities through a variety of processes, such as enteric methane (CH_4_) production from ruminant livestock. In the United States, enteric CH_4_ emissions from beef cattle comprise approximately 2% of total U.S. GHG emissions (EPA, 2024). Beef cattle production in the United States expands across a range of climatic zones from subtropical to temperate, and the threat of increased thermal load of finishing beef cattle is a sustainability challenge. Methodologies that have been proposed for feedlot beef cattle to address risks posed by a warming climate include 1) utilization of growth-promoting technologies (GPT) (Reichhardt et al., 2023), 2) increasing the proportion of *Bos indicus* (BI) breeds that are more adapted to hot and humid climates (Boyles and Riley, 1991; Ferrell and Jenkins, 1998; Elzo et al., 2009), and 3) modification of facilities and feed management practices (Reinhardt and Brandt, 1994; Mader, 2003; Mader and Davis, 2004).

The use of GPT is suggested to be one of the most effective mitigation strategies to decrease GHG emissions from beef production per unit of feed consumed and product produced (Boadi et al., 2004; Monteny et al., 2006; Stackhouse et al., 2012). The main GPT used in finishing operations are antibiotics, ionophores, anabolic implants, and beta-adrenergic ligands (Stackhouse-Lawson et al., 2013). Antibiotics can be used to improve cattle health, as well as control and prevent disease, whereas ionophores are typically fed to improve feed efficiency (Goodrich et al., 1984; Stackhouse et al., 2012). Anabolic implants employ estrogenic (estradiol or zeranol) and androgenic (testosterone or trenbolone acetate) components to improve feed intake, nitrogen utilization, and protein deposition (Bryant et al., 2010; Drouillard, 2018). Moreover, ractopamine hydrochloride, a beta-adrenergic agonist, can be fed to feedlot cattle during the last 28 to 42 d of the feeding period to reduce ammonia emissions, promote protein deposition and improve feed efficiency, leading to increased hot carcass weight (HCW) (Mersmann, 1998; Bryant et al., 2010; Johnson, 2014). Despite potential productivity and efficiency improvements resulting from the use of GPT, previous research has reported that different cattle breeds may respond differently to GPT (Reichhardt et al., 2023). Therefore, it is important to quantify differences in the effect of GPT to best inform future breed selection and management decisions to ensure the resiliency of beef production systems.

Breed comparison research, observing primarily growth performance and carcass attributes of *Bos taurus* (BT) and BI sub-species of cattle were explored extensively throughout the 20th century as cattle producers were importing BI cattle to the United States (Black et al., 1934; Sanders, 1980; Paschal et al., 1995). Nearly half a century since the foundational and preliminary comparisons of BI and BT cattle, an eminent need exists to proactively address the climate adaptivity and resiliency of beef cattle in a rapidly changing climate. However, preceding research has observed 3 primary drawbacks to increasing the proportion of BI genetics in the fed cattle industry, which are: 1) decreased growth performance (Adams et al., 1982; Boyles and Riley, 1991), 2) poorer carcass attributes (Boyles and Riley, 1991; Ferrell and Jenkins, 1998) and 3) more excitable cattle temperament (Voisinet et al., 1997), relative to BT. Moreover, despite their global prominence, BI cattle are underrepresented in current CH_4_ emissions evaluations from ruminant species (Della Rosa et al., 2022). With limited data available representative of current cattle type and technology comparing the growth performance, carcass characteristics, and gas flux, a need exists for comparative feedlot research of BI and BT steers to aid in future beef industry sustainability assessments.

Therefore, the objective of the current experiment was to evaluate enteric gas flux of CH_4_, CO_2_, O_2_, and H_2_, growth performance, and carcass characteristics of BI and BT sub-species managed with and without the use of GPT in summer conditions. The authors hypothesized that BT steers and steers managed with GPT would exhibit greater growth ­performance, primarily due to greater feed intake and feed efficiency compared to BI steers and steers not managed with GPT. Predicted difference in growth performance will influence enteric gas flux emissions, where cattle that consume more feed are expected to exhibit greater CH_4_ emissions; thus, BT steers are expected to have greater CH_4_ emissions than BI steers.

## Materials and Methods

This experiment was conducted at the Colorado State University Agricultural Research, Education, and Development Center experiment station in Fort Collins, Colorado, from April to October 2023, consisting of one continuous 180 d feeding period divided into 2 phases. All procedures involving animals were approved by the Colorado State University Institutional Animal Care and Use Committee (Protocol No. 3712). All animals evaluated in the current study were sourced in two lots by Five Rivers Cattle Feeding, where lot 1 was comprised of BT steers sourced from Montana and lot 2 of BI steers sourced from Texas. Steers of BI origin were allotted 10 d to acclimate to the research facility prior to study initiation; whereas BT steers were allotted 4 d. Acclimatization was determined based on differences in climatic conditions between the research facility and source location for each lot.

### Animals and experimental design

Animals consisted of 100 BI (Brahman) yearling steers (initial body weight (IBW) = 342 kg ± 31 kg) and 100 BT (Angus) yearling steers (IBW) = 341 kg ± 21 kg). Steers of each sub-species were randomly assigned to a management treatment, managed with the use of GPT (TRT) or without the use of GPT (CON), in a 2 × 2 factorial arrangement of treatments. Steers in TRT received an in-ear anabolic implant on d 0 (100 mg trenbolone acetate/14 mg estradiol benzoate; Synovex Choice, Zoetis, Parsippany, NJ, USA) and d 84 (200 mg trenbolone acetate/28 mg estradiol benzoate; Synovex Plus, Zoetis). Steers in TRT also received an in-feed ionophore (35 g/ton DM basis; monensin, Rumensin, Elanco, Greenfield, IN, USA) and antibiotic (7 g/ton DM basis; tylosin, Tylan, Elanco) from d 0-180. Also, TRT steers received a beta-adrenergic agonist during the last 42 d of the feeding period (27 g/ton DM basis; ractopamine hydrochloride, Actogain, Zoetis), allowing for a 2 d withdrawal period prior to harvest. The CON steers did not receive any of the above-listed GPT.

Regardless of management and sub-species, all cattle were vaccinated with 2 mL Bovi-Shield GOLD 5 (Bovine rhinotracheitis-virus diarrhea-parainfluenza 3-respiratory syncytial virus vaccine, Zoetis), 2 mL Ultrachoice 8 (clostridial vaccine, Zoetis), Dectomax (doramectin, Zoetis), and orally drenched with Valbazen (albendazole, Zoetis) within 48 h period post arrival to the feedlot. All cattle received a radio frequency identification tag (Allflex, USA Inc., Dallas, TX, USA) placed in the left ear of each animal on the same processing date.

Steers were fed for 180 d in 2 consecutive phases. In Phase 1 (d 0 to 83), steers were housed for 84 d in research pens (7 m × 40 m), where cattle were blocked by weight and randomly assigned to a pen, where the pen was considered the experimental unit (10 steers/pen; 5 pens/treatment). Each pen was equipped with a concrete feed bunk (30 cm/animal), a 3 m × 7 m concrete bunk pad, and an automatic water tank (model no. C250, Cancrete Cattle Waterer, Advanced Agri-Direct Inc., York, NE, USA). In Phase 2 (d 84 to 180), cattle were moved to 1 of the 4 Climate Smart Research Pens (15 m × 43 m) for the remaining 96 d of the experimental period. Each Climate Smart Research Pen housed 50 steers/pen, equipped with 1 GreenFeed automated head chamber system (AHCS, C-Lock, Rapid City, SD, USA), 5 SmartFeed (SF, C-Lock, Rapid City, SD, USA) bunk systems, 2 automatic water tanks, a concrete bunk pad (15 m × 3 m), and a metal roof (15 m × 3 m) covering the SFs, AHCS, and approximately 7% of the pen surface. Treatments were randomly assigned to a Climate Smart Research Pen (1 pen/treatment), and the experimental unit was considered the individual animal (50 steers/treatment).

Throughout the experimental period, steers were monitored daily by trained personnel who evaluated fresh feed and water allocation, cattle health, locomotion, and clinical signs of disease. Steers exhibiting signs of respiratory disease were removed from the pen to assess rectal body temperature and body weight (BW). Cattle with body temperatures greater than 39.4°C were classified as clinically ill. All clinically ill steers were treated according to the appropriate treatment protocol prescribed by a licensed veterinarian and immediately returned to their pen of origin. Cattle that were managed under the CON strategy that were treated were documented and not included in the analysis of data in order to simulate a production typical scenario representative of a “natural” labeled beef program where cattle treated with antibiotics are removed from the production system. Following the completion of the experimental period, a total of 6 steers were removed from data analysis due to injury or illness: 1 BI TRT, 1 BI CON, and 4 BT CON.

### Feed management and intake

All steers were transitioned to the final finishing diet after a 21 d adaptation from a starter diet to a finishing diet, including two intermediate step-up diets (Table 1). Feed samples were collected weekly, dried at 65°C for a minimum of 48 h to determine dry matter (DM), and ground to pass through a 2 mm screen (Wiley Mill, Model 4; Arthur H. Thomas Co., Philadelphia, PA, USA) and preserved at room temperature. After the completion of the experimental period, feed samples were composited and analyzed at a commercial laboratory using a wet chemistry package (Ration Balancer Plus with Gross Energy and Starch, Dairy One, Ithaca, NY, USA). Acid detergent fiber analysis was conducted following ANKOM Technology Method 12, where 0.5 g of a feed sample was individually weighed, placed in a filter bag, and digested in an ANKOM DELTA Digestion Unit for 75 minutes in 2 L of acid detergent fiber solution. Samples were rinsed three times with boiling water for 5 minutes in filter bags followed by a 3-minute acetone soak and drying at 105°C for 2 hours. Neutral detergent fiber analysis was conducted following ANKOM Technology Method 13, where 0.5 g of a feed sample was individually weighed, placed in a filter bag, and digested in an ANKOM DELTA Digestion Unit for 75 minutes in 2 L of neutral detergent fiber solution. At the start of digestion, 4 ml of Alpha Amylase and 20 g sodium sulfite were added. Sample were then rinsed three times with boiling water for 5 minutes, with 4 ml Alpha Amylase added to the first two rinses. Water rinses were followed by a 3-minute acetone soak and drying at 105°C for 2 hours. Analysis of lignin was determined following ANKOM Technology Method 9 using a DaisyII Incubator. For crude protein analysis, dried feed samples were ground to 1 mm and combusted using a CN628 Carbon/Nitrogen Determinator. Determination of ash used the Association of Official Agricultural Chemists Official Method 942.05-Ash for Animal Feed. Gross energy was determined using bomb calorimetry.

**Table 1.** Dietary ingredients and analyzed chemical composition of the diets

Item	Diet			
	Starter	Step 1	Step 2	Finish
**Ingredient, % DM**				
Steam-flaked corn	30.0	44.0	55.0	65.0
Corn silage	50.0	40.0	30.0	20.0
Distillers grains w/ solubles	13.5	9.5	8.5	7.0
Molasses-based supplement	3.3	3.3	3.3	4.0
Mineral and vitamin supplement	3.2	3.2	3.2	4.0
**Chemical Composition, DM basis** ^1^				
DM, %	48.2	49.2	59.9	65.7
CP, %	15.0	14.8	13.4	14.2
ADF, %	13.3	11.4	8.6	8.9
NDF, %	19.9	17.8	14.3	14.3
Lignin, %	2.7	2.8	2.5	2.3
Starch, %	45.1	49.8	57.0	57.6
Ash, %	6.8	5.3	4.0	4.6
TDN, %	77.0	79.0	82.0	82.0
NE_m_, Mcal/kg	1.89	1.95	2.02	2.03
NE_g_, Mcal/kg	1.25	1.31	1.37	1.38
GE, Mcal/kg	4.49	4.45	4.51	4.44

1 Dietary composition was determined via wet chemistry.

During Phase 1, slick-bunk management was utilized to manage feed delivery to the feed bunk, where feed bunks were evaluated at approximately 07:00 hours daily to estimate orts and adjust feed calls to ensure *ad libitum* access to feed. Feed intake data were averaged daily on a steer/pen basis. Daily feed allocation per pen was divided by the number of steers/pen, averaged by week, and multiplied by the percent DM of the total mixed ration to determine average daily dry matter intake (DMI) on a weekly basis. During Phase 2, individual animal feed intake was determined utilizing SF bunk systems, and *ad libitum* bunk management was utilized to manage feed delivery (approximately 2.5% orts). A ratio of approximately 1 feeder per 10 steers was maintained throughout the experimental period. The SF bunks were evaluated at approximately 07:00 hours daily where the manufacture’s online dashboard was utilized to report orts. Fresh feed was delivered once daily to achieve no less than approximately 5 kg remaining in each feeder the following day at 07:00 hours. All cattle underwent a 7 d acclimation period to the SF bunks before the initiation of data collection. Feed intake data collected during the acclimation period were not used in data analysis. The SF bunks were calibrated at the beginning and end of the experimental period and every 7 d during the experimental period. Following the termination of the experimental period, raw feeding events were downloaded from the manufacturer’s online software. Feeding events that were flagged by the manufacturer (unallocated feed or nonsensical eating rate) exceeded 3,600 s in duration and/or were greater than 10 kg mass difference were omitted from data analysis. Feeding events were summed by day and averaged by week to determine weekly as-fed feed intake. As-fed feed intake was converted to DMI by multiplying by the percent DM of the total mixed ration. To determine the average DMI for each phase, weekly DMI was averaged over the feeding period phase. During Phase 2 of the experimental period, DMI from alfalfa pellet drops from the AHCS was added to the total mixed ration daily intake to determine total daily DMI. In addition to total daily DMI in Phases 1 and 2, DMI as a percentage of BW was calculated for each phase using equation 1.


(1) $$\mathrm{DMI},\;\%\;BW=\frac{\mathrm{Dry}\;\mathrm{matter}\;\mathrm{intake},\;kg}{BW,\;kg}\;\times 100.$$

### Growth performance

Steers were individually weighed using a hydraulic squeeze chute (Model no. SHEW10497, Silencer, Moly Manufacturing Inc., Lorraine, KS, USA) equipped with an electronic weighing system (ID5000, Tru-Test Inc., Mineral Wells, TX, USA) on 2 consecutive days at beginning of the experiment (d − 1 and 0) to determine IBW and at the end (d 179 and 180) of the experimental period to define final BW (FBW). In addition to IBW and FBW, steers were weighed every 28 d during the experimental period. Experimental mid-point BW (MBW), collected on d 84, was used as the FBW for Phase 1 and IBW for Phase 2. Weight data collected were reduced by 4% to account for digestive tract fill (i.e. shrunk body weights [SBW]; NASEM, 2016). The SBW from each time point, every 28 d, was used to obtain cattle average daily gain (ADG) by subtracting the SBW of the previous time point from the SBW of the current time point and then dividing by the number of days between the 2 weight collection periods.

### Gas flux

Each Climate Smart Research Pen contained one AHCS for the collection of gas fluxes for CH_4_, CO_2_, H_2_, and O_2_. Steers were exposed to the AHCS during an acclimation period of 7 d before the initiation of data collection. The headbox extension panels (i.e. wings) were placed on the AHCS system on d 3 of the acclimation period. On d 5 of the acclimation period, cattle panels were used to create an alley in front of the AHCS to ensure only 1 steer at a time had access to the AHCS. Measurements collected during the acclimation period were not included in the data analysis. Throughout the study, steers were allowed to visit the AHCS system up to 6 visits/d, with a minimum of 4 h between each visit. The AHCS was programmed to allow consumption of up to 6 drops of alfalfa pellets per visit (approximately 38 g as fed/drop), with drops occurring every 30 s while a steer was utilizing the AHCS system. These configuration settings encouraged an even distribution of visits per steer throughout the day, capturing the diurnal variation in gas flux and ensuring a steer remained at the AHCS for a minimum duration of 2 min during gas flux sample collection (Vargas et al., 2024).

To ensure proper functioning and performance of the AHCS, CO_2_ recovery tests were performed at the beginning, end, and every 28 d throughout the experimental period to gravimetrically calibrate the AHCS. Additionally, zero and span calibrations of the CH_4_, CO_2_, H_2_, and O_2_ gas analyzers were performed every 3 d via an autocalibration system located onboard the AHCS. Raw collection data were validated by the AHCS manufacturer, which included checking head proximity, visit length, and airflow, as well as providing corrections for wind. Data were excluded when the length of the visit was less than 2 min, greater than 8 min, and the airflow was less than 26 L/s (Arthur et al., 2017; Gunter and Beck, 2018). Steers were excluded from the analysis of data if the total number of visits collected throughout Phase 2 was less than 40 (Beck et al., 2024). Of the 194 steers, excluding the 6 steers that were removed from the trial for health-related reasons, 166 steers met the filtering requirements to be included in the analysis of gas flux data analysis, 36 BI CON, 42 BI TRT, 44 BT CON, and 44 BT TRT. For the steers that met the filtering requirements, the average number of gas flux visits per treatment were as follows: 140 visits/steer for BI CON, 146 visits/steer for BI TRT, 117 visits/steer for BT CON, and 164 visits/steer for BT TRT.

In addition to the collection of CH_4_, CO_2_, H_2_, and O_2_ gas flux, CH_4_ as a proportion of gross energy intake (GEI)(Y_m_), CH_4_ as a proportion of DMI (MY), and CH_4_ as a proportion of ADG (EI) in Phase 2, was calculated using equations 2, 3, and 4, respectively.


(2) $$Ym,\;\%\;\mathrm{GEI}=\;\frac{\mathrm{Gaseous}\;\mathrm{Energy}\;\mathrm{Loss},\;\mathrm{mcal}/g}{\mathrm{GEI},\;\mathrm{mcal}/g}\times 100$$

(3) $$MY,\frac{g}{kg}=\frac{{CH}_{4},\;g}{\mathrm{DMI},\;kg}$$

(4) $$EI,\frac{g}{kg}=\frac{{CH}_{4},\;g}{\mathrm{ADG},\;kg}$$

Furthermore, CH_4_ as a proportion of carcass gain in Phase 2 (EI_cg_) was calculated using equation 5 derived from Tatum et al. (2012) and equation 6.


(5) $$\mathrm{Phase}\;2\;\mathrm{Estimated}\;\mathrm{Carcass}\;\mathrm{Gain},\;kg=\mathrm{Final}\;\mathrm{HCW}-(\mathrm{Estimat}ed\;\mathrm{Beginning}\;\mathrm{HCW}=0.2598\times {\mathrm{IBW}}^{1.1378})$$

(6) $${EI}_{CG},\;\frac{g}{kg}=\frac{{CH}_{4},\;g}{\mathrm{Phase}\;2\;\mathrm{Estimated}\;\mathrm{Carcass}\;\mathrm{Gain},\;kg}$$

### Carcass characteristics

Following the collection of weights on d 180, steers were shipped to a commercial abattoir in Greeley, Colorado, approximately 48.3 km from the Colorado State University Agricultural Research, Education, and Development Center, for harvest. Harvest data was collected by a third party (Diamond T Livestock Services, Inc., Alliance, NE, USA). On the same day as slaughter, HCW and liver abscess prevalence (scored as 0, A−, A, and A+; Brown et al., 1975) were assessed. Carcasses were chilled for 24 h at −4°C. Approximately 24 h after slaughter, the carcasses were ribbed between the 12th and 13th ribs, and carcass data were collected via cameras, including 12th rib backfat thickness (FT), longissimus muscle area (LMA), and marbling score. Yield grade (YG) was calculated using the United States Department of Agriculture (USDA) YG equation with a standard kidney, pelvic, and heart fat percentage of 2.0 (USDA, 1997; Boykin et al., 2017). The USDA YG and quality grade (QG) for each carcass were assigned by a USDA grader at the commercial abattoir. Dressing percentage (DP) was calculated by dividing HCW by shrunk FBW and multiplying by 100.

### Environment

Environmental data was collected using the publicly available CoAgMET service to acquire ambient air temperature (°C; T_a_), relative humidity (%, RH), solar radiation, and wind speed data collected continuously throughout the experimental period from an on-site weather station (station no. ftc03) at the Colorado State University Agricultural Research, Education, and Development Center (40.65°N, −105°E) located 9.7 km northeast of Fort Collins at an elevation of 1,557.5 m. Temperature humidity index (THI) was calculated using T_a_ and RH in equation 7, adapted from Thom (1959).


(7) $$\mathrm{THI}=(0.8\times T_{a})+[\left(\mathrm{RH}÷100)\times (T_{a}-14.4\right)]+46.4$$

**Figure 1.**
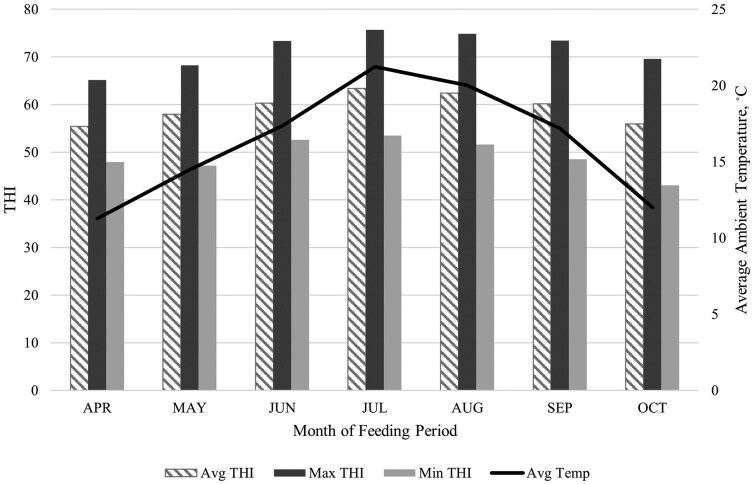
THI and average ambient temperature during the 180d feeding period in summer feedlot conditions: THI = (0.8 × T_a_) + [(RH/100) × (T_a_−14.4)] + 46.4 (Thom, 1959), where T_a_ is ambient temperature (°C), and RH is relative humidity (%); Min = minimum; Max = maximum; Avg = average.

Descriptive statistics are reported for weather station data collected during the 180 d feeding period (Table 2). An average ambient temperature of 16°C was exhibited, with an average minimum of approximately 4°C and an average maximum of 31°C. July was the hottest month, with an average temperature of 21°C, exhibiting a minimum ambient temperature of approximately 10°C and a maximum temperature of approximately 36°C during the month. Relative humidity averaged 58%, with an average minimum of approximately 10% and an average maximum of 95% during the experimental period. Solar radiation averaged approximately 240 W/m^2,^ and wind speed averaged approximately 2 m/s during the experimental period. Moreover, THI averaged 59, with an average minimum of 49 and an average maximum of 72 (Figure 1).

**Table 2.** Descriptive statistics of environmental variables measured during the 180 d feeding period in summer feedlot conditions

**Month** ^1^	Environmental variable							
	**T_a_,** °C			RH, %			WS, m/s	SR, W/m^2^
	Min	Max	Mean	Min	Max	Mean	Mean	Mean
APR	2.56	22.11	11.24	0.19	0.76	0.41	3.3	290.22
MAY	1.87	26.77	14.48	0.07	0.97	0.63	2.5	237.27
JUN	8.31	33.07	17.30	0.09	0.99	0.69	2.1	262.44
JUL	9.77	35.61	21.20	0.11	0.99	0.60	1.9	257.23
AUG	7.09	34.81	19.89	0.15	0.97	0.64	1.7	238.79
SEP	3.55	32.40	17.19	0.09	0.98	0.54	1.8	218.74
OCT	-3.54	28.40	12.01	0.09	0.97	0.52	2.1	174.80

1 Month of 180 d feeding period were APR = April, MAY = May, JUN = June, JUL = July, AUG = August, SEP = September, OCT = October.

### Statistical analysis

The experimental design of Phase 1 of the experimental period was a randomized complete block design with a 2 × 2 factorial arrangement of treatments. Data were analyzed with R (R Core Team, 2021, v. 4.4.1) software using the lmer() function within the lme4 package to construct a linear mixed-effects model. The model diagnostics included testing for normal distribution of the error residuals via visual assessment of the histogram and Q-Q plot for each variable, as well as formally conducting the Shapiro-Wilk test, homogeneity of variance via visual assessment of the residual vs. fitted values plot, and independence among observations which was ensured during the study design. The assumptions were adequately held.

For Phase 1, the fitted model was:


$$Y_{\mathrm{ijkl}}=µ+S_{i}+T_{j}+{\left(ST\right)}_{ij}+W_{k}+B_{l}+E_{\mathrm{ijkl}}$$

where Y_ijkl_ is the response variable; S_i_ is the fixed effect of sub-species; T_j_ is the fixed effect of treatment; (ST)_ij_ is the interaction between sub-species and treatment; W_k_ is the fixed effect of initial body weight of the phase; B_l_ is the random effect of block; and E_ijkl_ is the experimental error.

The experimental design of Phase 2 of the experimental period was a completely randomized design with a 2 × 2 factorial arrangement of treatments. Data were analyzed with R (R Core Team, 2021, v. 4.4.1) software using the aov() function within the base R package to construct a linear effects model. The model diagnostics included testing for normal distribution of the error residuals, homogeneity of variance, and independence among observations using methodologies previously described. The assumptions were adequately held.

For Phase 2, the fitted model was:


$$Y_{\mathrm{ijk}}=µ+S_{i}+T_{j}+{\left(ST\right)}_{ij}+W_{k}+E_{\mathrm{ijk}}$$

where Y_ijk_ is the response variable; S_i_ is the fixed effect of sub-species; T_j_ is the fixed effect of treatment; (ST)_ij_ is the interaction between sub-species and treatment; W_k_ is the fixed effect of initial body weight of the phase; and E_ijk_ is the experimental error.

Categorical data distributions were analyzed by logistic regression. The model was constructed using the glm() function of the base R package. Least-squares means were derived using the emmeans package in R, with Tukey’s HSD adjustment. For Phase 1 pen was the experimental unit, and for Phase 2 individual animal was the experimental unit. Differences were declared significant at *P* ≤ 0.05, and tendencies were discussed between 0.05 < *P* ≤ 0.10.

## Results

### Growth performance

In Phase 1, IBW did not differ (*P* ≥ 0.75) by cattle sub-species or management treatment (Table 3). However, FBW differed (*P <* 0.01) by cattle sub-species and management treatment, with BT steers weighing more than BI steers and steers managed with the use of GPT having greater FBW than CON. There was a difference (*P* < 0.01) in ADG between cattle sub-species and management treatment, with BT steers having greater ADG compared to BI steers, and steers managed without the use of GPT having less ADG than steers managed with GPT. Even though DMI did not differ (*P* = 0.35) by management treatment, BT steers had greater (*P* < 0.01) DMI than BI steers on a total daily basis and as a percentage of BW. Furthermore, G:F also differed (*P* < 0.01) by cattle sub-species and management treatment, with BT steers having greater feed efficiency than BI steers and TRT steers having greater feed efficiency than CON steers.

**Table 3.** Growth performance of finishing *Bos taurus* and *Bos indicus* steers managed with and without the use of growth-promoting technologies during Phase 1 (0 to 83 days) in summer feedlot conditions

Item	*Bos indicus*		*Bos taurus*		SEM	S	T	S×T
	CON	TRT	CON	TRT				
*n*, pen	5	5	5	5	–	–	–	–
IBW, kg	343	341	342	340	12.4	0.90	0.75	0.98
FBW, kg	417	445	497	521	3.9	<0.01	<0.01	0.65
ADG, kg	0.90	1.23	1.84	2.14	0.046	<0.01	<0.01	0.67
DMI, kg/d	6.77	7.07	9.86	9.85	0.222	<0.01	0.35	0.49
DMI_BW_, % of BW	1.77	1.80	2.35	2.29	0.048	<0.01	0.74	0.39
G:F	0.132	0.174	0.192	0.230	0.0029	<0.01	<0.01	0.45

In Phase 2, IBW differed (*P* < 0.01) by cattle sub-species and management treatment, with BT steers exhibiting greater IBW than BI steers and TRT steers exhibiting greater IBW than CON steers (Table 4). Additionally, FBW exhibited a sub-species × treatment interaction (*P* < 0.01), where the effect of using GPT on FBW was greater for BT steers than BI steers. Similarly, ADG had a sub-species × treatment interaction (*P* < 0.01), with the use of GPT resulting in a greater magnitude of difference in BT steers than BI steers. Moreover, DMI differed (*P* < 0.01) by sub-species and treatment, with BT steers having greater DMI than BI steers and steers managed with the use of GPT exhibiting greater DMI than steers managed without GPT. However, as a percentage of BW, DMI exhibited a sub-species × treatment interaction where the direction of the response to the use of GPT differed (*P* = 0.01) by sub-species. The use of GPT resulted in a greater magnitude of difference in DMI as a percentage of BW for BI steers, whereas in BT steers, DMI as a percentage of BW did not differ. Furthermore, G:F exhibited a sub-species × treatment interaction (*P* = 0.02), with the use of GPT exhibiting a greater magnitude of difference for BT steers vs. BI steers.

**Table 4.** Growth performance of finishing *Bos taurus* and *Bos indicus* steers managed with and without growth-promoting technologies during Phase 2 (84 to 180 days) in summer feedlot conditions

Item	*Bos indicus*		*Bos taurus*		SEM	S	T	S×T
	CON	TRT	CON	TRT				
*n*, animal	49	49	46	50	–	–	–	–
IBW, kg	420	443	495	519	5.9	<0.01	<0.01	0.95
FBW, kg	564^c^	604^b^	594^b^	652^a^	4.1	<0.01	<0.01	<0.01
ADG, kg	0.99^c^	1.40^b^	1.30^b^	1.91^a^	0.043	<0.01	<0.01	<0.01
DMI, kg/d	7.45	8.30	9.91	10.40	0.167	<0.01	<0.01	0.20
DMI_BW_, % BW	1.43^b^	1.54^b^	1.86^a^	1.85^a^	0.029	<0.01	0.48	0.01
G:F	0.134^b^	0.170^a^	0.129^b^	0.182^a^	0.0043	0.53	<0.01	0.02

a,b,c Means in the same row within main effect that do not have a common superscript letter differ, *P* ≤ 0.05.

### Carcass characteristics

Similar to FBW in Phase 2, HCW exhibited a sub-species × treatment interaction (*P* = 0.05; Table 5). The magnitude of difference in HCW between CON and TRT steers was greater for BT steers than for BI steers. Moreover, DP differed (*P* ≤ 0.04) by cattle sub-species and management treatment, favoring BI steers and steers that were not managed with GPT. The backfat thickness of the carcass did not differ (*P* ≥ 0.18) by cattle sub-species or management treatment. Additionally, LMA differed (*P* < 0.01) by cattle sub-species and management treatment. Steers managed with the use of GPT exhibited greater LMA than those managed without GPT, and BT steers had greater LMA than BI steers. Calculated YG did not differ (*P =* 0.18) by cattle sub-species but tended (*P* = 0.06) to be greater for CON steers compared to TRT steers. Marbling score exhibited a sub-species × treatment interaction (*P* < 0.01), where steers managed with GPT exhibited a greater decrease in marbling score than CON steers for BT when compared to BI steers, which did not exhibit a difference in marbling score between treatments.

**Table 5.** Carcass data from *Bos taurus* and *Bos indicus* steers managed with and without the use of growth-promoting technologies following a 180-day feeding period in summer feedlot conditions

Item^1^	*Bos indicus*		*Bos taurus*		SEM	S	T	S×T
	CON^2^	TRT^2^	CON^2^	TRT^2^				
*n*, animal	49	49	46	50	–	–	–	–
HCW, kg	361^c^	381^b^	375^b^	405^a^	2.9	<0.01	<0.01	0.05
DP, %	63.9	63.2	63.1	62.0	0.28	0.04	<0.01	0.58
FT, cm	1.22	1.13	1.22	1.21	0.052	0.96	0.18	0.40
LMA, cm^2^	82.5	88.2	86.3	91.1	1.18	<0.01	<0.01	0.66
Calculated YG	3.05	2.85	2.98	2.98	0.087	0.61	0.06	0.19
Marbling score	344^c^	329^c^	600^a^	483^b^	15.1	<0.01	<0.01	<0.01

1 FT = back fat measured between the 12th and 13th rib after 24 h chill; marbling score scale: 300 = slight, 400 = small, 500 = modest, 600 = moderate.

2 Experimental management treatment.

a,b,c Means in the same row within main effect that do not have a common superscript letter differ, *P* ≤ 0.05.

When assessing YG distribution, the percent distribution of YG differed (*P* < 0.01) by sub-species for YG 1, 2, and 3 but did not differ (*P* = 0.14) by management treatment (Table 6). Carcasses of BI steers had a greater proportion of YG 1 and 2 when compared to BT. However, BT carcasses had a greater proportion of YG 3 carcasses than BI. For the proportion of YG 4 carcasses, a sub-species × treatment interaction was observed (*P* = 0.02), where BT TRT carcasses had a greater proportion of YG 4 carcasses compared to CON steers but did not differ by management treatment for BI steers. When observing the USDA QG carcass distribution, QG distribution differed (*P* < 0.01) by sub-species for USDA Prime, Choice, and Standard grades, as well as dark cutter carcass designations. Carcasses of BI steers had a greater (*P* < 0.01) proportion of USDA Standard QG. However, USDA Choice and USDA Prime QG carcasses were observed in greater (*P* < 0.01) proportions for BT than BI. Moreover, USDA Prime-graded carcasses also differed (*P* < 0.01) by treatment, with a greater proportion of CON carcasses grading USDA Prime than TRT for BT steers. Moreover, BI steers were observed to have a greater incidence of dark cutter carcasses than BT. The proportion of carcasses grading USDA Select had a sub-species × treatment interaction (*P* < 0.01).

**Table 6.** United State Department of Agriculture yield and quality grade distribution for *Bos taurus* and *Bos indicus* steers managed with and without the use of growth-promoting technologies following a 180-day feeding period in summer feedlot conditions

Item^1^	*Bos indicus*		*Bos taurus*		SEM	S	T	S×T
	CON^2^	TRT^2^	CON^2^	TRT^2^				
*n*, animal	49	49	46	46	–	–	–	–
**USDA YG, %**								
YG 1	20.4	20.4	0.0	0.0	5.76	<0.01	1.00	1.00
YG 2	46.9	57.1	28.3	20.0	7.13	<0.01	0.87	0.17
YG 3	28.6	22.4	69.6	70.0	6.78	<0.01	0.65	0.59
YG 4	4.1^ab^	0.0^b^	2.2^b^	10.0^a^	4.24	0.14	0.52	0.02
**USDA QG, %**								
Prime	0.0	0.0	26.1	6.0	6.47	<0.01	<0.01	1.00
Choice	20.4	10.2	73.9	80.0	6.47	<0.01	0.70	0.13
Select	40.8^a^	44.9^a^	0.0^b^	14.0^b^	7.11	<0.01	0.11	<0.01
Standard	32.7	38.8	0.0	0.0	6.96	<0.01	0.53	1.00
Dark Cutter	6.1	6.1	0.0	0.0	3.42	<0.01	1.00	1.00

1 Quality grade marketing category determined by expert marbling score: USDA Prime = Slightly Abundant^0^ to Abundant^99^; USDA Choice = Small^0^ to Moderate^99^; USDA Select = Slight^0^ to Slight^99^; USDA Standard = Traces^0^ to Traces^99^.

2 Experimental management treatment.

a,b Means in the same row within main effect that do not have a common superscript letter differ, *P* ≤ 0.05.

Liver abscess prevalence was assessed, observing a difference (*P* < 0.01) in the proportion of non-abscessed livers between management treatments, with TRT steers having a greater proportion of non-abscessed livers than CON steers (Table 7). For A-minus liver scores, abscess prevalence differed (*P* = 0.02) by treatment, with CON having a greater proportion of A-minus scored livers compared to TRT. For A-scored livers, CON tended (*P* = 0.08) to have a greater proportion of A-scored livers than TRT. However, A-plus liver scores did not differ (*P* = 0.11) by treatment. Total abscess prevalence (0 vs. A-, A, and A+) differed (*P* < 0.01) by management treatment, with TRT steers exhibiting less abscessed livers than CON steers. Liver scores did not differ (*P* ≥ 0.27) by cattle sub-species.

**Table 7.** Liver abscess prevalence data from *Bos taurus* and *Bos indicus* steers managed with and without the use of growth-promoting technologies following a 180 day feeding period in summer feedlot conditions

Item^1^	*Bos indicus*		*Bos taurus*		SEM	S	T	S×T
	CON^2^	TRT^2^	CON^2^	TRT^2^				
*n*, animal	49	49	46	50	–	–	–	–
**Liver abscess prevalence, %**								
0	81.6	93.9	73.9	92.0	6.47	0.34	<0.01	0.86
A−	2.0	0.0	6.5	0.0	3.64	0.27	0.02	1.00
A	6.1	0.0	4.4	2.0	3.42	0.95	0.08	0.22
A+	10.2	6.1	15.2	6.0	5.30	0.56	0.11	0.65
Total abscess prevalence	18.4	6.1	26.1	8.0	6.47	0.34	<0.01	0.86

1 Liver abscess prevalence where: 0 = non-abscessed liver; A− = one or two small abscesses; A = two to four small, active abscesses; A+ = one or more large, active abscesses, including any open and adhered abscesses.

2 Experimental management treatment.

### Gas flux

Absolute emissions of CH_4_, in g/d, differed (*P* < 0.01) by cattle sub-species, with BT steers emitting more CH_4_ than BI steers (Table 8). Gas flux of CO_2_ and O_2_ also differed (*P* < 0.01) by cattle sub-species and management treatment. Moreover, H_2_ emissions had a sub-species × treatment interaction (*P* < 0.01), where H_2_ decreased when steers were managed with GPT for BT steers but increased when steers were managed with GPT for BI steers. Furthermore, the yield of CH_4_, as a proportion of gross energy intake (Y_m_), was greater (*P* < 0.01) for BT steers than for BI steers. Similar to Y_m_, MY was greater (*P* < 0.01) for BT steers compared to BI steers, meaning more CH_4_ was emitted per unit of feed intake by BT steers than BI steers. Conversely, EI exhibited a sub-species × treatment interaction (*P* < 0.05), with steers of both sub-species managed with GPT exhibiting a lesser EI than CON counterparts; however, BT steers exhibited a greater reduction in EI when compared to BI steers. Similarly, EI_CG_ had a sub-species × treatment interaction (*P* = 0.02), where the use of GPT decreased CH_4_ emissions per unit of carcass gain, but a greater reduction in EI_CG_ was observed for BT steers between CON and TRT than for BI steers.

**Table 8.** Gas flux of finishing *Bos taurus* and *Bos indicus* steers managed with and without the use of growth-promoting technologies during Phase 2 (84 to 180 days) in summer feedlot conditions

Item^1^	*Bos indicus*		*Bos taurus*		SEM	S	T	S×T
	CON^2^	TRT^2^	CON^2^	TRT^2^				
*n*, animal	36	42	44	44	–	–	–	–
AHCS visits, *n*	140	146	117	164	–	–	–	–
DMI, kg/d	7.51	8.33	9.96	10.47	0.198	<0.01	<0.01	0.32
ADG, kg	0.97^c^	1.35^b^	1.32^b^	1.93^a^	0.050	<0.01	<0.01	<0.01
CH_4_, g/d	123	133	179	180	5.5	<0.01	0.13	0.28
CO_2_, g/d	7,062	7,604	9,927	10,244	143.0	<0.01	<0.01	0.32
O_2_, g/d	5,069	5,455	6,754	7,215	92.3	<0.01	<0.01	0.61
H_2_, g/d	0.78^c^	1.03^b^	1.45^a^	1.07^bc^	0.076	<0.01	0.22	<0.01
Y_m_, % GEI	4.92	4.81	5.43	5.12	0.190	<0.01	0.21	0.52
MY, g CH_4_/kg DMI	16.5	16.1	18.1	17.1	0.63	<0.01	0.21	0.53
EI, g CH_4_/kg ADG	131.1^a^	101.9^b^	140.7^a^	92.8^b^	5.98	0.05	<0.01	0.05
EI_CG_, g CH_4_/kg CG	1.72^b^	1.47^b^	2.04^a^	1.48^b^	0.084	<0.01	<0.01	0.02

1 Y_m_ = CH_4_ emitted as a percent of GEI.

2 Experimental management treatment.

a,b,c Means in the same row within main effect that do not have a common superscript letter differ, *P* ≤ 0.05.

## Discussion

### Growth performance

Historically, BI breeds have received pushback from the beef feedlot and packing industries, primarily in regard to poor growth performance and carcass characteristics (Huffman et al., 1990). Historically the primary benefit of BI cattle has been their use in crossbreeding programs in the South and Southwest regions of the United States, where the resulting offspring exhibit enhanced adaptability to the hot, humid climatic conditions (Paschal et al., 1995). Therefore, climatic conditions in which BI cattle have evolved differ from the climatic conditions of the experimental location in the present study. However, to garner more information regarding the sustainability tradeoffs of increasing the proportion of BI genetics in the U.S.-fed cattle industry as an adaptation strategy for feeding cattle in increasingly warmer climates, the experimental location offers the ability to evaluate thermal extremes. Moreover, it would be beneficial to understand more about their feedlot growth performance compared to BT cattle, considering management with and without the use of GPT, to acknowledge any deviations from past observations while also incorporating current market niches (Cartwright, 1980; Boyles and Riley, 1991).

Ferrell and Jenkins (1998) documented that steer genotype can influence the rate of growth and G:F. When considering BI and BT comparative research trials, previous observations have reported that BT cattle consumed greater quantities of feed with greater feed efficiency than BI counterparts, generally corresponding to greater growth rates (Arthur et al., 1994; Ferrell and Jenkins, 1998; Elzo et al., 2009). Observations by Butler et al. (1956b) reported the growth performance advantage of BT cattle vs. BI cattle when they were fed grain-based diets. It has been proposed that differences in growth performance between BT and BI cattle fed grain-based diets could be an outcome of evolution since BI cattle originated in tropical climates where feeding systems generally depend on poor-quality forage resources (Chaokaur et al., 2015). This idea was supported by research from Arthur et al. (1994), who observed that when growing steers were raised in different nutritional environments, BI steers gained more BW than BT counterparts on poor-quality pasture, but the inverse was observed when steers were placed on a good-quality pasture. Therefore, the previously described observations regarding greater rates of growth for BT steers relative to BI steers when fed a greater-quality diet would apply to the results of the present study where BT steers outperformed BI steers on a grain-based diet. The observed cattle sub-species difference is a necessary production challenge to consider in the application of increasing the proportion of BI genetics in feedlot systems where grain-based diets are commonly fed.

In Phase 1 of the current study, BT steers had approximately 35% greater DMI and 26% greater DMI as a percentage of BW, which resulted in 61% greater ADG and 32% greater G:F ratio on average compared to BI steers. The observed increase in ADG resulted in an average 17% increase in FBW in the first 84 d of the feeding period for BT steers when compared to BI steers. Growth performance observations reported in Phase 1 of the present study were consistent with observations in Phase 2. Differences in feed efficiency in Phases 1 and 2 between BT and BI steers could be attributed to differences in protein vs. fat carcass accretion influenced by the stage of animal growth and proximity to mature body size in each phase (Owens et al., 1995). As an animal deposits more fat relative to protein, they may become less efficient due to fat deposition requiring more energy than protein deposition (Owens et al., 1995). Bidner et al. (2002) concluded that mature body size varies amongst breeds of cattle, with Angus reaching mature body size at lighter body weight than BI-influenced cattle, requiring fewer days on feed to reach a common backfat thickness. Additionally, Frish and Vercoe (1977) explained that as BI cattle evolved under poorer nutritional conditions, they were naturally selected for slower fasting metabolism (good survival ability) corresponding to less voluntary feed intake, resulting in decreased growth rates under good nutritional conditions relative to BT cattle. Available literature supports the suggestion that BI cattle, in general, have reduced maintenance requirements relative to BT cattle (van der Merwe and van Rooyen, 1980; Solis et al., 1988). It has been proposed that differences in maintenance requirements, among other factors like feed intake habits and behavior, gastrointestinal tract capacity, and confinement sensitivity, may, in part, contribute to differences in feed efficiency between BI and BT cattle (Butler et al., 1956b; Cartwright, 1980). Future research regarding differences in feeding behavior amongst breeds would provide further insight to aid in the identification of feed management practices for different breeds when fed in confinement.

According to NASEM (2016), beef steers managed without the use of anabolic implants are predicted to have 6% less feed intake than steers managed with anabolic implants, and steers managed without monensin are predicted to have 3% greater intake than steers managed with monensin. Interestingly, when specifically considering BT steers in the present study, while Phase 1 observed DMI as a percentage of body weight was greater than predicted (2.3 vs. 2.2; equation 10-4; NASEM, 2016), Phase 2 DMI on a percentage of body weight basis was slightly less than predicted (1.9 vs. 2.0; equation 10-4; NASEM, 2016). When evaluating CH_4_ emission classification and ranking of non-implanted and implanted steers in the finishing phase of beef production, data from Vargas et al. (2025) yielded an average DMI as a percentage of BW of 1.7, when steers were fed a similar finishing diet to the present study, with net energy of gain being 1.32 Mcal/kg DM. However, Vargas et al. (2025) utilized a stocking rate of approximately 5 steers per SF bunk systems. In Phase 2 of the present study, steers were managed for *ad libitum* intake where bunk competition was equal for all treatments with approximately 10 steers per SF bunk system across treatments. The stocking rate for SF bunk systems varies in the literature but is generally between 4 and 8 hd/bunk systems, with the manufacturer’s general recommendation between 7 and 8 hd/bunk system for feedlot steers (Holder et al., 2022; Fuerniss et al., 2023; Vargas et al., 2024). In the present study, the authors acknowledge an aggressive stocking rate, but do not believe stocking rate inhibited *ad libitum* feed intake due to evaluation of daily orts, which averaged approximately 6 kg per bunk at 07:00 hours. Additionally, the ratio of bunk use to total possible bunk time was calculated, yielding a ratio of 0.43 for BT steers. The application of this ratio can be interpreted as there was approximately 14 h/SF bunk/d of excess bunk time when bunks were not used but could have been used. However, appropriate stocking rate methodologies should be investigated for SF bunk systems to improve consistency in equipment utilization across research environments and management methodologies to allow for improved comparisons among the literature. These methodologies are critical to evaluate due to the relationship between level of feed intake and CH_4_ emissions (Johnson and Johnson, 1995).

Beyond the influence of cattle sub-species, previous research by Reichhardt et al. (2023) observed that different cattle breeds responded differently to the use of GPT. This observation was demonstrated in the present study. In Phase 1, steers managed with GPT had 21% greater ADG and 22% greater G:F compared to steers managed without GPT. However, the use of GPT did not affect DMI or DMI as a percentage of BW in Phase 1. Conversely, in Phase 2, when steers were managed with GPT, TRT steers consumed, on average, 7% more feed per day than CON steers. Moreover, when DMI was reported as a percentage of BW in Phase 2, the use of GPT increased DMI by 7% for BI steers, while BT steers were observed to have a 0.5% decrease in DMI. Furthermore, in Phase 2, ADG was greater for steers managed with GPT, with the use of GPT resulting in a 34% increase in ADG for BI steers and a 38% increase in ADG for BT steers. Greater ADG in TRT steers corresponded to a 7% increase in FBW of BI steers and 9% increase in FBW for BT steers relative to CON counterparts.

In a meta-analysis of modern technologies commonly used in beef production, Wileman et al. (2009) reported that cattle that were implanted with anabolic implants had a 17% improvement in ADG and a 9% improvement in G:F when compared to non-implanted cattle. Lean et al. (2014) conducted a meta-analysis investigating growth performance characteristics of feedlot cattle supplemented with a beta-adrenergic agonist and reported ractopamine hydrochloride supplementation resulted in approximately an 8 kg increase in BW and 0.19 kg/d increase in ADG with no substantial difference in DMI compared to non-supplemented cattle. Similarly, increases in ADG from 11.1% to 27.4% in steers supplemented with ractopamine hydrochloride at 200 mg for the final 28 d of the feeding period have been previously reported (Laudert et al., 2005a; Schroeder et al., 2005b). Ractopamine hydrochloride supplementation has consistently been demonstrated to increase BW, ADG, and G:F in beef cattle when compared to non-supplemented counterparts (Scramlin et al., 2010; Genther-Schroeder et al., 2016; Bryant et al., 2020). Moreover, Duffield et al. (2012) conducted a meta-analysis investigating the effect of monensin supplementation on beef cattle growth performance and observed a 6.4% increase in G:F influenced by a 3% decrease in DMI and a 2.5% increase in ADG. A lack of research exists considering the synergistic effects of managing cattle using multiple GPT. Despite the use of multiple GPT in the present study and the inability to identify how each GPT may be influencing the observed growth performance, this research clearly demonstrates that the use of GPT resulted in greater FBW, ADG, and G:F in feedlot steers. However, the present study also highlights that cattle of differing sub-species do not elicit the same growth performance response when using GPT, highlighting that GPT use may be more valuable to use in certain cattle sub-species from a cattle husbandry standpoint compared to others.

### Carcass characteristics

In general, BI cattle have been reported to have less desirable carcass quality attributes, primarily marbling, when compared to BT cattle breeds, resulting in scrutiny from the fed cattle and beef packing sectors of the beef industry (Huffman et al., 1990). Preceding observations would suggest that BT cattle generally exhibit greater HCW, YG, and marbling scores when compared to BI contemporaries (Adams et al., 1982; Lunt et al., 1985; Paschal et al., 1995; Gruber et al., 2007). In the present study, the effect of GPT use on HCW differed between cattle sub-species. Steers of the BI sub-species that were managed with GPT had an observed 5% greater HCW than counterparts managed without GPT. However, steers of the BT sub-species managed with GPT exhibited an 8% greater HCW than counterparts managed without GPT. Observations of the present study are consistent with preceding research investigating GPT use. Bryant et al. (2010) observed that steers receiving an anabolic implant exhibited greater HCW when compared to non-implanted steers. Moreover, Bryant et al. (2020) observed that cattle receiving ractopamine hydrochloride exhibited greater HCW than non-supplemented cattle. Furthermore, in the present study, BT steers had a 4% greater LMA than BI steers, and the use of GPT increased LMA, with TRT steers having a 6% greater LMA than CON. Previously documented observations have exhibited that both anabolic implants and beta-adrenergic agonists increase LMA compared to non-implanted and non-supplemented cattle, respectively (Bryant et al., 2010; Bryant et al., 2020).

The effect of GPT on marbling score differed by sub-species, with BT CON steers obtaining marbling scores 22% greater than BT TRT steers, but no difference was observed for marbling scores for BI steers between treatments. However, despite a greater decrease in marbling scores between CON and TRT BT steers, BT TRT steers still yielded greater marbling scores than BI steers. Huffman et al. (1990) reported that when the percentage of BI influence was 50% or greater in feedlot cattle, smaller marbling scores and subsequent poorer quality grades were observed relative to BT steers. In the present study, the majority of BI carcasses were observed to grade USDA Standard and USDA Select, whereas the majority of BT carcasses were observed to grade USDA Choice or USDA Prime. Additionally, BI carcasses exhibited a greater proportion of dark cutter grades than BT. When evaluating Brahman steers that received an anabolic implant, Smith et al. (2007) observed an average marbling score of 390.3, corresponding to the Select USDA quality grade. This observation is concurrent with observations of the present study, with BI steers that were receiving GPT exhibiting an average marbling score of 329, corresponding to the USDA Select quality grade. Ultimately, despite both cattle sub-species demonstrating an increase in HCW and LMA with the use of GPT, observations suggest that the cattle sub-species did not respond to the same magnitude to the use of GPT. Furthermore, BI steers demonstrated to have less intramuscular fat deposition, regardless the use of GPT or not, when compared to BT steers.

Liver abscess prevalence is not only an economic concern in the fed cattle industry but also an animal welfare concern that impacts the sustainability of beef cattle production systems (Baier et al., 2020). In the present study, tylosin phosphate was supplemented in the diet of TRT steers. When considering total liver abscess prevalence (0 vs. A-, A, and A+), CON steers that did not receive tylosin phosphate had approximately three times greater liver abscess prevalence than TRT steers. Similarly to the present study, Müller et al. (2018) observed that 21.4% of cattle that did not receive supplemental tylosin phosphate presented with liver abscesses, whereas only 7.8% of cattle that received supplemental tylosin phosphate had liver abscesses. Data from Müller et al. (2018) demonstrated that cattle that did not receive tylosin phosphate had approximately three times greater liver abscess prevalence than cattle receiving tylosin phosphate, which is similar to the present study. Moreover, the DP of cattle is an economic factor of great importance (Butler et al., 1956b). In previous comparative studies, reports vary regarding the outcome of DP between cattle sub-species (BT vs. BI). Some reports suggest that BT cattle have shown an advantage in DP (Adams et al., 1982), while others suggest BI cattle have shown an advantage in DP (Black et al., 1934; Butler et al., 1956a, 1956b). Black et al. (1934) reported that steers that were approximately half Brahman had DP between 2% and 4% greater than BT steers. In the present study, BI steers had a DP 2% greater than BT steers. Differences in digestive tract size between BI and BT steers offer a plausible explanation for differences in DP between sub-species, as BI steers have been observed to have smaller digestive tracts relative to BT steers (Black et al., 1934). However, the present study observed that BT CON steers had greater DP than TRT steers, whereas previous research suggested that the use of GPT should result in greater DP (Bryant et al., 2010; Ross et al., 2021). Future research should evaluate economic tradeoffs in carcass quality and quantity for BT and BI steers to understand the margin of relative profitability that could be anticipated if the proportion of BI cattle in the U.S.-fed cattle industry increases in the future.

### Gas flux

Increasing atmospheric concentrations of GHG and their contribution to global warming have led scientists to examine their sources of origin (Johnson and Johnson, 1995; Shibata and Terada, 2010). Methane is a short-lived but potent GHG that has a variety of biogenic and anthropogenic sources, one of which is through enteric CH_4_ production from wild and domesticated ruminant species. In an assessment of global ruminant CH_4_ emissions measurements, it was observed that most cattle studies assessed BT cattle, with only 12% of cattle studies evaluating BI cattle (Della Rosa et al., 2022). However, BI cattle represent more than half the global cattle population and are the predominant cattle sub-species in tropical and subtropical climatic zones around the world (Utsunomiya et al. 2019). Given the genomic, phenotypic, and feed consumption differences, it is logical to postulate that there may be differences in CH_4_ emissions between BI and BT sub-species of cattle. Collection of data that is representative of the global cattle population is critical to evaluate in order to improve GHG emissions inventories and aid in more accurately representing the contribution of CH_4_ at a global and national scale from cattle (Della Rosa et al., 2022).

In the present study, absolute CH_4_ emissions differed by sub-species, with BT steers producing approximately 33% more CH_4_, in g/d, than BI steers. Therefore, from an absolute emissions standpoint, the present study has observed that BI steers emitted less CH_4_ than BT steers. Nonetheless, many factors influence CH_4_ emissions from cattle, requiring further investigation in the context of these two sub-species. Some factors that have been extensively described include the level of feed intake, type and quality of carbohydrates in the diet, feed processing, additives (e.g. lipids, ionophores), and alterations in the ruminal microflora (e.g. defaunation) (Johnson and Johnson, 1995; Shibata and Terada, 2010; Beauchemin et al., 2022). Therefore, it is logical to postulate that the difference in absolute CH_4_ emissions between BT and BI steers in the current study were influenced by feed intake differences. However, to the authors’ knowledge, this is the first study of its kind and future comparative CH_4_ evaluations of BI and BT cattle may consider investigation of the rumen microbial community and rate of digestion to isolate factors influencing CH_4_ production differences between cattle sub-species beyond feed intake in order to identify potential interventions and mitigation opportunities.

A complexity that exists in the realm of animal science regarding gas flux measurement is the interpretation of absolute gas production vs. gas production yield per unit of input or output (Beauchemin et al., 2022). Beyond enteric CH_4_ emissions from beef cattle being an environmental sustainability concern, these emissions are also problematic with respect to energy utilization efficiency (Chaokaur et al., 2015). According to Johnson and Johnson (1995), cattle typically emit 6% of their gross energy intake (GEI) as eructated CH_4_, but emissions may vary between 2% and 12%. National inventories for enteric CH_4_ are calculated in accordance with the Intergovernmental Panel on Climate Change guidelines, where the CH_4_ conversion factor, also known as Y_m_, is a critical metric used to assess the potential extent of global warming and to estimate total enteric CH_4_ emissions (Houghton et al., 1996; Steinfeld et al., 2006; Gerber et al., 2013). The Intergovernmental Panel on Climate Change Tier 1 methodology involves multiplying the cattle population by a standard emissions factor, whereas the Tier 2 methodology involves a more complex approach (Beauchemin and McGinn, 2006). For cattle, Tier 2 emission factors are estimated based on the GEI and the corresponding standardized Y_m_ based on the production system (grazing vs. confinement; IPCC, 2006). The Intergovernmental Panel on Climate Change guidelines for a Tier 2 approach set a default Y_m_ of 3% for feedlot cattle fed steam-flaked corn in North America, a value generated predominately utilizing BT gas flux measurements (IPCC, 2006).

In the present study, Y_m_ ranged from approximately 4.8% to 5.4%. Between sub-species, BT steers were observed to have approximately 8% greater Y_m_ when compared to BI steers. Johnson et al. (1993) extrapolated chamber measurements to typical diets at common levels of intake that occur across U.S. beef cattle herds and suggested that Y_m_ varies from approximately 5.8% to 6.5% for all categories and classes except for the unique feedlot situation, where grain-based diets are fed, in which typical Y_m_ may drop to approximately 3%. The present study if 3% Y_m_ was used to represent emissions from this population of beef cattle fed a concentrate-based diet during the feedlot phase of production, emissions would be underestimated. Moreover, the present study observed that when considering MY, BT steers had approximately 8% greater MY than that of BI. Schutt et al. (2009) observed that BI cattle have a greater efficiency of energy capture relative to BT. Therefore, evolution differences in the digestive anatomy and physiology between the sub-species may be a plausible reason why Y_m_ and MY are less for BI steers relative to BT. However, when CH_4_ was expressed as the mass of CH_4_ emitted per unit gain, the impact of managing steers with or without GPT resulted in a greater magnitude of difference in EI for BT steers than BI steers. For BT steers, forgoing the use of GPT resulted in 41% greater EI, whereas for BI steers, this difference was 25%. Moreover, when considering EI_CG_, the use of GPT resulted in a 16% decrease in CH_4_ per unit carcass gain in BI steers and a 32% decrease in BT steers. These observations emphasize the halo effect that occurs throughout value-based systems, like agri-food systems, where the sustainability of a product is attributed to the perceived health and safety benefit, which necessitates education and communication of the sustainability advantages of using GPT in cattle production systems. Moreover, the contradiction of absolute emissions vs. emissions efficiency conceptualizes the complexity of interpreting the sustainability of beef cattle production. From strictly an environmental standpoint, limiting total emissions is of high priority. However, when simultaneously considering topics of food security and production efficiency, balancing yield and environmental impact is crucial (Place, 2024).

## Conclusion

The purpose of this study was to evaluate the growth performance and gas flux of BT and BI steers managed with and without the use of GPT in summer feedlot conditions. From a growth performance perspective, BT steers grew faster and ate more, finishing at heavier FBW when compared to BI steers. Moreover, GPT increased DMI and ADG, resulting in greater feed efficiency, lending to heavier FBW. Additionally, observations demonstrated that the effect of GPT differed by sub-species for ADG, G:F, FBW, HCW, and marbling score. For gas flux, BI steers emitted less CH_4_ than BT steers and had a lesser yield of CH_4_ as a proportion of GEI and DMI due to the consumption of less feed. However, GPT reduced GHG emissions when expressed per unit of gain or beef produced, with a greater decrease demonstrated between treatments in BT vs. BI steers. Furthermore, BT steers produced greater quality carcasses, highlighting a limitation of BI steers in a consumer demand-driven industry.

*Conflict of interest statement.* Authors, A.K.S.-H., K.R.S.-L., J.J.V., E.C.M., W.A.S., M.A.S., E.N.B., R.K.C., A.M.S., J.P.R., S.E.P., and P.H.V.C., declare no real or perceived conflicts of interest. Author T.C.B is Director of Nutrition, Research, and Analytics for Fiver Rivers Cattle Feeding. Five Rivers Cattle Feeding provided the cattle utilized in this research but were not involved in data collection, analysis, or interpretation of results.

## Abbreviations

ADF: acid detergent fiber
ADG: average daily gain
AHCS: automated head chamber system
BI: *Bos indicus*
BT: *Bos taurus*
CH_4_: methane
CO_2_: carbon dioxide
CON: cattle managed without growth-promoting technology
CP: crude protein
DM: dry matter
DMI: dry matter intake
DMI_BW_: dry matter intake according to the body weight
DP: dressing percentage
EI: emissions intensity
EI_cg_: emissions intensity-carcass gain
FBW: final body weight
FT: backfat thickness
G:F: gain to feed ratio
GE: gross energy
GEI: gross energy intake
GHG: greenhouse gas
GPT: growth-promoting technology
H_2_: hydrogen
HCW: hot carcass weight
IBW: initial body weight
LMA: longissimus muscle area
MBW: mid-point body weight
Max: maximum
Min: minimum
MY: methane yield
NDF: neutral detergent fiber
NE_m_: net energy for maintenance
NE_g_: net energy for gain
O_2_: oxygen
QG: quality grade
RH: relative humidity
S: main effect cattle sub-species
S × T: interaction between cattle sub-species and management treatment
SBW: shrunk body weight
SF: SmartFeed
SR=: solar radiation
T: main effect management treatment
T_a_: ambient temperature
TDN: total digestible nutrients
THI: temperature humidity index
TRT: cattle managed with growth-promoting technology
US: United States
USDA: United States Department of Agriculture
WS=: wind speed
YG: yield grade
Y_m_: yield of methane
